# Cervical myelopathy caused by atlantoaxial instability in a patient with an os odontoideum and total aplasia of the posterior arch of the atlas: a case report

**DOI:** 10.1186/1752-1947-6-171

**Published:** 2012-06-28

**Authors:** Tadanori Ogata, Tadao Morino, Masayuki Hino, Hiromasa Miura

**Affiliations:** 1 Spine Center, Ehime University Hospital, Tohon-city, Ehime, 791-0295, Japan; 2 Department of Bone and Joint Surgery, Ehime University Graduate School of Medicine, Tohon-city, Ehime, 791-0295, Japan

## Abstract

**Introduction:**

Congenital hypoplasia of the atlas has rarely been reported. Myelopathy caused by the complete absence of the posterior arch of the atlas has not been reported. This case report describes the diagnosis and successful treatment of a myelopathy due to the complete absence of the posterior arch of the atlas.

**Case presentation:**

A 59-year-old Japanese man experienced pain in his nuchal region with progressive spasticity, numbness and hypesthesia in his upper and lower limbs. Deep tendon reflexes in his upper and lower limbs were increased. The complete absence of the posterior arch of the atlas and atlantoaxial instability were found in a roentgenogram. Magnetic resonance imaging detected high signal intensity on T2-weighted images in his spinal cord at the level of cervical vertebrae 1 to 2. Our patient underwent posterior occipito-C4 fixation with pedicle screws. After the operation, the pain in his nuchal region disappeared and his symptoms of myelopathy improved. Only slight numbness of his upper limbs remained.

**Conclusions:**

This is the first report of myelopathy due to the complete absence of the posterior arch of the atlas.

## Introduction

Congenital absence of the posterior arch of the atlas is a rare anomaly. Currarino *et al*. developed a classification system of congenital defects of the posterior arch of cervical vertebra 1 (C1) [1]. Type A denotes the failure of posterior midline fusion, with a small gap or fissure. Type B involves unilateral clefts and defects range from a small gap to a complete absence of the hemi-arch including the posterior tubercle. In type C, there are bilateral clefts and a bony defect present in the lateral aspect of the arch bilaterally with preservation of the most dorsal part of the arch. Type D refers to the absence of the posterior arch with persistent posterior tubercle. In type E, the entire arch is absent, including the posterior tubercle.

To date, there have been 11 cases of total aplasia (type E) [1-7]. Most of them were incidentally found by X-ray examination, and some of them caused non-specific neck pain. Until now, there have been no reports of patients with type E defects of the C1 posterior arch showing neurological deficits. We describe a case of cervical myelopathy caused by atlantoaxial instability in a patient with total aplasia of the C1 posterior arch.

## Case presentation

A 59-year-old Japanese man presented with a six-month history of numbness of his right hand and dull pain in his nuchal region. He had no history of cervical trauma. Three months before the first visit to our hospital, numbness had spread over all four of his limbs. No muscle weakness was observed; however, our patient showed gait ataxia because of the spasticity of his lower extremities. In a physical examination, bilateral hypesthesia (soft touch and pin prick) and a marked increase of the deep tendon reflexes in his upper and lower limbs were observed.

**Figure 1:**
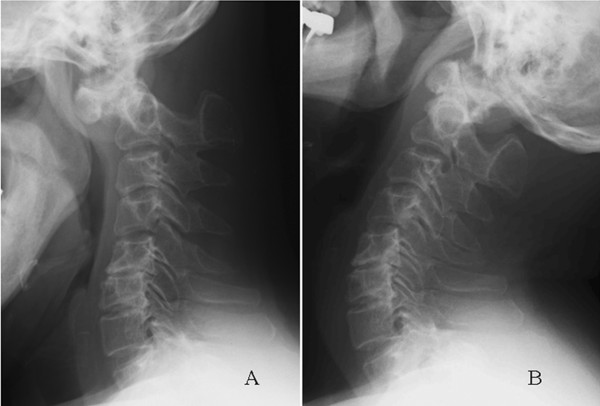
**Atlantoaxial instability with absence of the posterior arch of the atlas.** Lateral radiographs of the cervical spine during (**A**) flexion and (**B**) extension show atlantoaxial instability.

**Figure 2:**
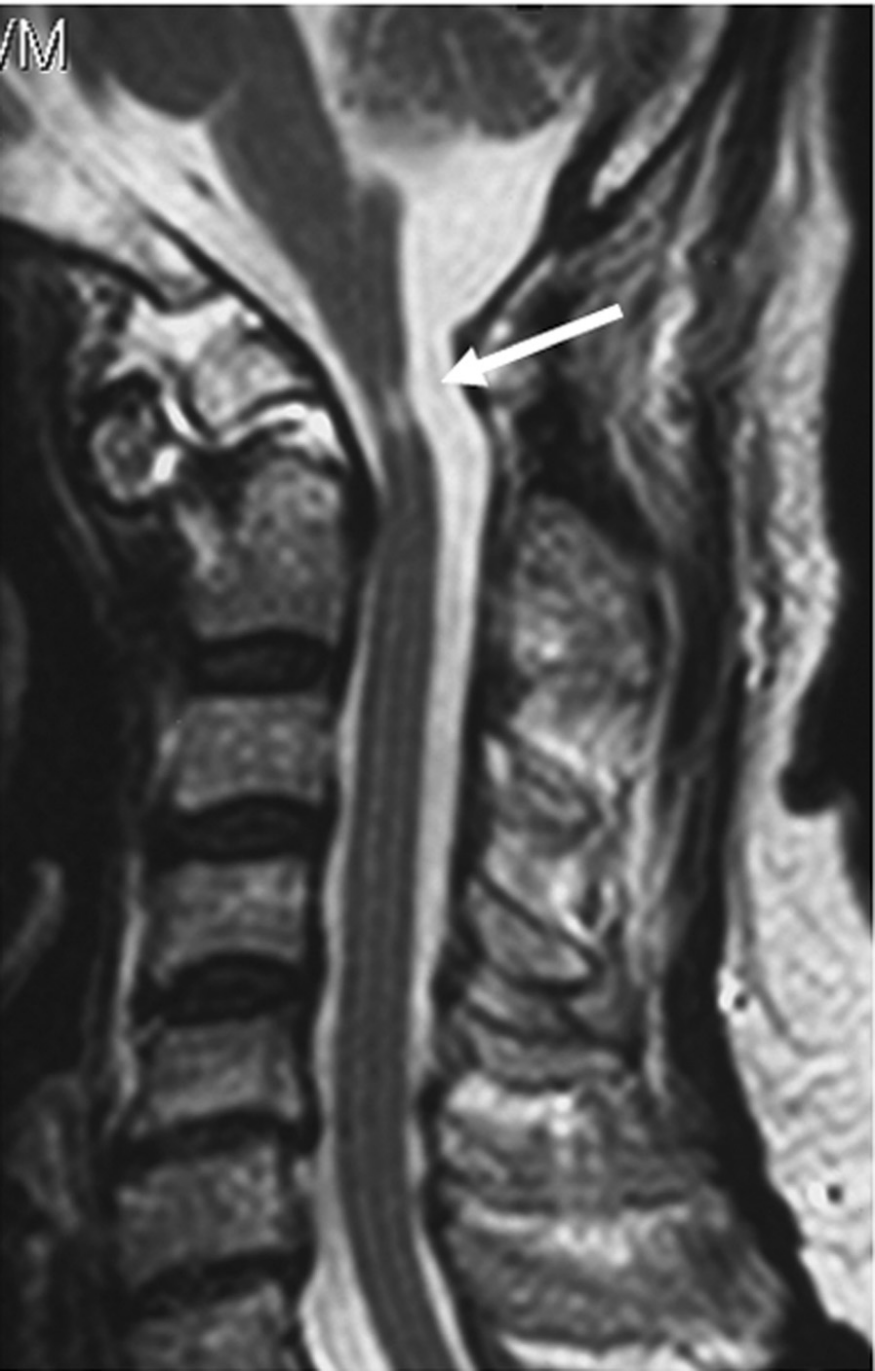
**Preoperative sagittal T2-weighted magnetic resonance imaging.** Atrophy of the spinal cord with myelomalacia, which was observed as a high intensity lesion (arrow), was seen.

**Figure 3:**
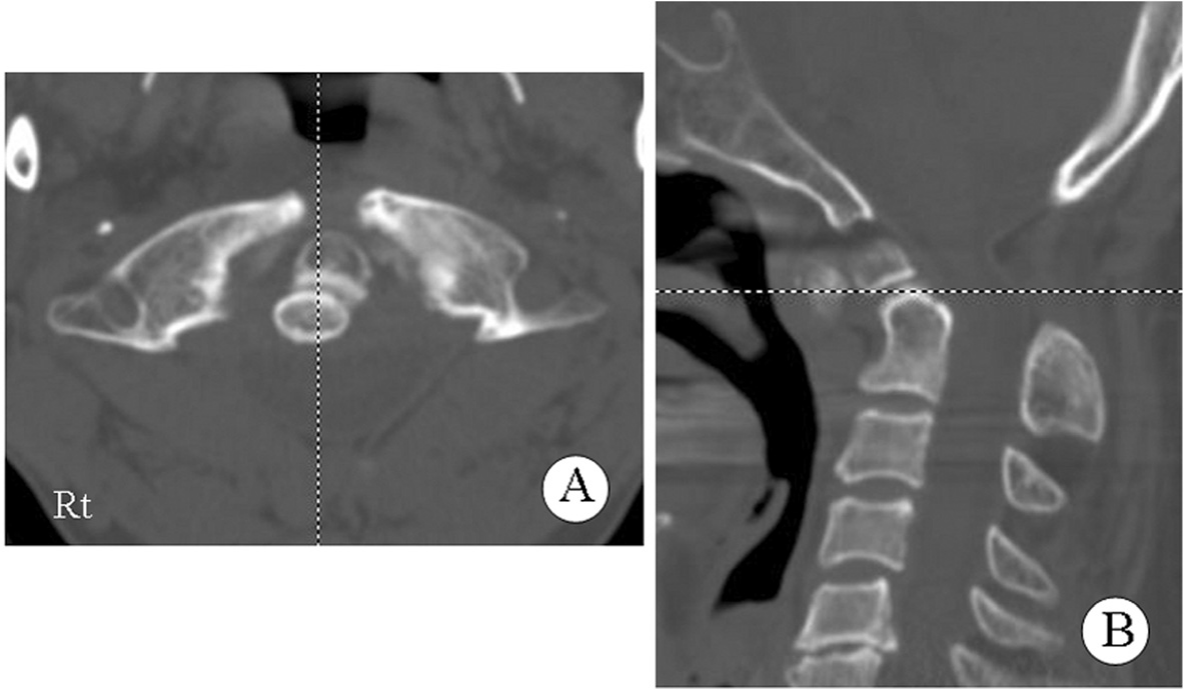
**Computed tomography images of the cervical spine.** (**A**) Complete absence of the posterior arch of the atlas with an anterior cleft was observed. (**B**) The sagittal plane of the three-dimensional computed tomography scan shows the os odontoideum.

**Figure 4:**
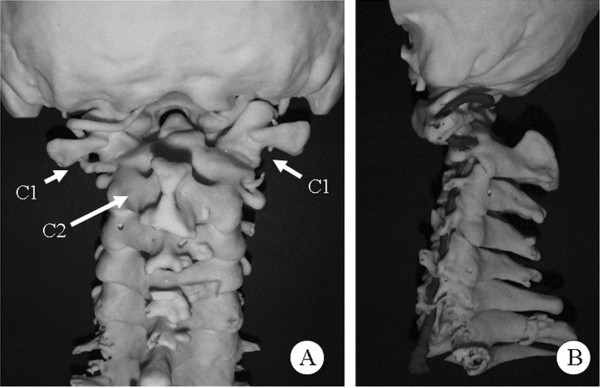
**Stereoscopical model of the cervical spine based on three-dimensional computed tomography data.** (**A**) The view from the posterior direction. (**B**) The view from the lateral direction. Complete absence of the C1 posterior arch and hypertrophy of the spinous process of the axis were observed.

Lateral X-ray films showed the absence of the posterior arch of the atlas along with atlantoaxial instability (Figure 1). The atlantodental interval was 9.62 mm in a flexion position (Figure 1A) while no interval was observed in an extension position (Figure 1B). Sagittal magnetic resonance imaging revealed atrophy of his spinal cord and myelomalacia that was observed as a T2-high signal intensity area (Figure 2) at the C1 level. Computed tomography (CT) showed the complete absence of the C1 posterior arch (Figure 3A) and an os odontoideum (Figure 3B). A cleft in the C1 anterior arch was also observed (Figure 3A): the two lateral masses were completely separated on both sides. A real model of his cervical spine was produced based on data from three-dimensional CT with contrast dye for the evaluation of the vertebral artery (Figure 4: produced by LEXI Co., Tokyo, Japan). Figure 4A shows the view from the posterior direction and Figure 4B shows the view from the lateral direction. Complete absence of the C1 posterior arch and hypertrophy of the spinous process of the axis were observed.

Our patient underwent posterior occipito-C4 fixation with pedicle screws. Our patient was placed in a prone position in a Mayfield head clamp. At the beginning of the operation, his neck was slightly flexed in order to expose the surface of the laminae. A midline incision was made with subperiosteal exposure of his occiput and cervical spine. His neck position was then changed to a neutral position. We did not observe any bony elements between his occipital bone and C2 lamina. His occipital plate was fixed with two bicortical screws. Pedicle screws were inserted bilaterally into the C3 and the C4 vertebrae and unilaterally into the right pedicle of C2. Since his left vertebral artery ran through the pedicle of C2 (high-riding transverse foramen), screws were not inserted into the left C2 pedicle in order to avoid vertebral artery injury. Then his neck position was changed to a moderate extension position under control of fluoroscopy to achieve a good atlantodental interval. Rod fixation was performed between his occipital plate and the C4 screws. Rigid fixation was achieved by this procedure. A corticocancellous graft harvested from his posterior iliac crest was placed on the decorticated occipital bone and C2 lamina and fixed to his occipital bone with a biodegradable poly-L-lactate screw (Fixsorb-MX; Takiron, Osaka, Japan). Residual bone chips were grafted on the surface of the decorticated lamina (C2 to C4) and fixed with fibrin glue.

**Figure 5:**
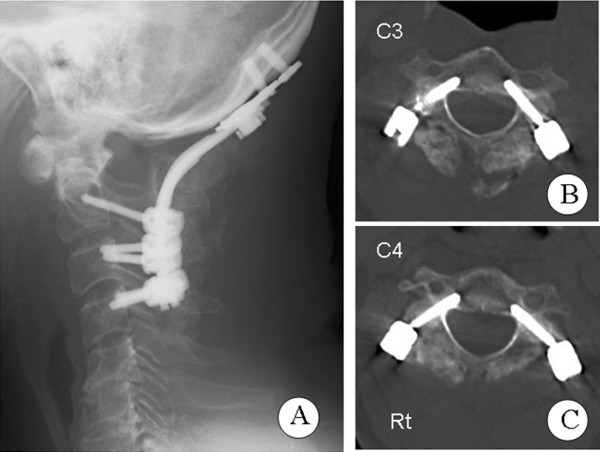
**Postoperative images.** (**A**) Lateral radiographs of the cervical spine and computed tomography images of the (**B**) C3 and (**C**) C4 vertebrae.

Our patient’s posture was managed using a cervical orthosis (Ortho collar, ARIZONO Orthopedic supplies co., Ltd., Kita-Kyusyu, Japan) for three months after surgery. Anteroposterior and lateral cervical radiographs were performed two, four and six weeks after the operation to check for loosening or dislocation of the implants and grafted bone. Three months after the operation, fusion between his occipital bone and C4 was assessed using lateral radiographs of his neck in flexion and extension position. Solid fusion was likely attained at this time (Figure 5A). The pedicle screws were positioned properly at C3 (Figure 5B) and C4 (Figure 5C). After the operation, the pain in his nuchal region disappeared and symptoms of his myelopathy improved. Only slight numbness of his upper limbs remained. Complete bony union of the grafted bone was observed in a CT scan performed six months after the operation. No recurrence of symptoms was observed during three years of follow-up after the operation.

## Discussion

The atlas has three primary ossification centers. The posterior arch of the atlas begins its ossification during the seventh week of intrauterine life, proceeding perichondrally from two centers located in the lateral masses. The two posterior halves are nearly fused at birth, except for several millimeters of cartilage. The neural arch completely fuses between the third and the fifth years of life. Ossification of the anterior arch involves one or two ossification centers, which extend in a posterolateral manner and fuse with the two lateral masses in the fifth year. In approximately 2% of the population, a fourth posterior ossification center forms the posterior tubercle between the two neural arches [8].

Congenital defects of the posterior arch of the atlas are uncommon. They are thought to be due to a failure of normal posterior arch chondrification processes. Clefts of the posterior arch of the atlas, mainly median, were found in 4% of 1,613 dissection specimens (Geipel cited in [3]). About 97% of posterior arch defects are type A. Congenital absence or hypoplasia of the posterior arch of C1 may also be associated with several diseases, such as the Arnold-Chiari malformation, gonadal dysgenesis, Klippel-Feil syndrome, and Turner and Down syndromes [2,9]. Samartzis *et al*. reported that congenital anomalies, such as aplasia or hypoplasia of the anterior or posterior of the atlas, increase the risk of neural injury in a patient with atlantoaxial subluxation and/or fixation [10].

Type A congenital defects are relatively common. We have encountered four cases of the type A defect. One case was found in a patient with Down’s syndrome with atlantoaxial instability, and the other three cases were found in patients with cervical spondylotic myelopathy. Recently, we have begun to use three-dimensional CT scans for preoperative radiographic examination. The latter three cases were found in the preoperative three-dimensional CTs. No clinical symptoms due to the type A defects were observed in our four cases. Patients with the type B defect also rarely show neurological deficits. We have encountered two cases of the type B defect. They were found incidentally, and no neurological abnormality due to the C1 defect was detected.

When an isolated tubercle is present (type C or D), a higher risk of spinal cord compression may be expected. Out of 26 reported cases of type C or D defects [1,3,9,11-14], 13 cases (50%) showed neurological deficits such as myelopathy, numbness, sensory disturbance and muscle weakness. Richardson *et al*. reported the mechanism by which neurological deficits occur during extension [11]. The isolated posterior tubercle, which is relatively loosely attached to the other posterior elements, moves anteriorly and traumatizes the dorsal spinal cord when the distance between the occiput and the spinous process of the axis is shorten by neck extension.

A total of 11 cases of type E defects have been reported [1-7]. Most of them were found incidentally. Three patients complained of slight neck pain, and the other patients showed no clinical symptoms. There have been no reports describing neurological symptoms in patients with type E anomalies. Geipel, cited by Dalinka *et al*., examined bony defects in the atlas by histology and found dense tracts of connective tissue crossing the gap. The connective tissue originated from the peritoneum and penetrated the bone [2]. Page *et al*. reported that stability at the atlantoaxial articulation results from the interaction of many ligaments and muscles, but is chiefly dependent on the transverse ligament of the atlas-odontoid-anterior arch relationship [4]. They showed that the transverse ligament between the lateral masses was intact despite complete aplasia of the posterior arch of C1. However, Schulze *et al*. reported atlantoaxial instability in a patient with type E aplasia, although the patient showed no clinical or neurological symptoms related to the anomaly of the atlas [3].

In our patient, serious atlantoaxial instability was seen during flexion-extension lateral radiography. The atlantodental interval was 9.62 mm in the flexion position while no interval was observed in the extension position (Figure 1). CT showed not only the complete absence of the C1 posterior arch, but also an os odontoideum and a cleft in the C1 anterior arch (Figure 3), that is, the two lateral masses were completely separated on both sides. Thus, there was no rigid bony structure at the C1 level. Three separate bone fragments - two lateral masses and an os odontoideum - were present between his occipital bone and the axis. It is possible that the os odontoideum shifted anteriorly together with the two lateral masses and the spinal cord was compressed by posterior fibrous tissue during cervical flexion.

Our patient’s symptoms of myelopathy remarkably improved after fixation between the occipital bone and C3 to C4 vertebrae. As far as we know, this is the first case of a type E defect in combination with an os odontoideum, anterior bony defects and myelopathy. Although our patient had a very rare anomaly, myelopathy possibly occurs in patients with a type E defect when another anomaly, such as os odontoideum, coexists.

In the operation, we decided to use relatively long fusion for the fixation of the atlantoaxial instability. Since the lateral halves of his C1 vertebra were completely separated, the atlas could not be used as a cranial anchor for atlantoaxial fixation. Moreover, we found a high-riding transverse foramen in the left side of his C2 vertebra. To avoid vertebral artery injury, only the right pedicle was used as an anchor. Finally, we decided to perform occipito-C4 fixations for this patient. Using pedicle screws, we successfully achieved solid fusion between the occipital bone and the C4 vertebra.

## Conclusions

We have reported a rare case of complete aplasia of the posterior arch of the atlas, which coexisted with an os odontoideum and a cleft in the anterior arch. Due to this unstable bony condition, atlantoaxial instability and myelopathy occurred. Fixation between the occipital bone and C4 successfully mitigated his clinical symptoms. The cervical pedicle screw technique appears to be useful for the long fixation of such unstable congenital upper cervical deformities.

## Consent

Written informed consent was obtained from the patient for publication of this case report and accompanying images. A copy of the written consent is available for review by the Editor-in-Chief of this journal.

## Competing interests

The authors declare that they have no competing interests.

## Authors’ contributions

TO performed the surgery and collected data from our patient. TM, MH and HM interpreted the data from our patient and were involved in drafting the manuscript. All authors read and approved the final manuscript.
